# MODSIDE: a motif discovery pipeline and similarity detector

**DOI:** 10.1186/s12864-018-5148-1

**Published:** 2018-10-19

**Authors:** Ngoc Tam L. Tran, Chun-Hsi Huang

**Affiliations:** Department of Computer Science and Engineering, University of Connecticut, Storrs, CT 06269 USA

**Keywords:** Binding sites, DNA motif, Motif detection tool, Motif discovery pipeline, Motif similarity detection, Motif clustering

## Abstract

**Background:**

Previous studies demonstrate the usefulness of using multiple tools and methods for improving the accuracy of motif detection. Over the past years, numerous motif discovery pipelines have been developed. However, they typically report only the top ranked results either from individual motif finders or from a combination of multiple tools and algorithms.

**Results:**

Here we present MODSIDE, a motif discovery pipeline and similarity detector. The pipeline integrated four de novo motif finders: ChIPMunk, MEME, Weeder, and XXmotif. It also incorporated a motif similarity detection tool MOTIFSIM. MODSIDE was designed for delivering not only the predictive results from individual motif finders but also the comparison results for multiple tools. The results include the common significant motifs from multiple tools, the motifs detected by some tools but not by others, and the best matches for each motif in the motif collection of multiple tools. MODSIDE also possesses other useful features for merging similar motifs and clustering motifs into motif trees.

**Conclusions:**

We evaluated MODSIDE and its adopted motif finders on 16 benchmark datasets. The statistical results demonstrate MODSIDE achieves better accuracy than individual motif finders. We also compared MODSIDE with two popular motif discovery pipelines: MEME-ChIP and RSAT peak-motifs. The comparison results reveal MODSIDE attains similar performance as RSAT peak-motifs but better accuracy than MEME-ChIP. In addition, MODSIDE is able to deliver various comparison results that are not offered by MEME-ChIP, RSAT peak-motifs, and other existing motif discovery pipelines.

**Electronic supplementary material:**

The online version of this article (10.1186/s12864-018-5148-1) contains supplementary material, which is available to authorized users.

## Background

Detecting binding site motifs can reveal the transcription factors that control the gene expression. Hence, numerous tools and methods have been developed for finding binding site motifs. Nevertheless, the results reported from different tools for an identical dataset are diverse. This is largely due to the fact that different tools implemented different algorithms and possesses unique features for discovering the motifs. Therefore, using multiple tools and methods has been suggested as it improved the accuracy of the motif detection [1–4]. The suggestion has inspired the development of several motif discovery pipelines. They can be standalone applications on standalone servers or pipelining Web servers. Recent development tends to be pipelining Web servers, which eliminate the complications of software installations and configurations required by standalone applications in order to serve more users via the Web. Another advantage is that it allows running multiple tools and methods at once on the same server and eliminates the manual runs of the same dataset on several different motif finders residing on the same standalone server or on several different Web servers.

The research community has seen several motif discovery pipelines such as W-ChIPMotifs [5], GimmeMotifs [6], CompleteMOTIFS [7], MEME-ChIP [8], RSAT peak-motifs [9], MotifLab [10], and Promzea [11] among many others. Generally, the pipelines incorporated multiple algorithms or tools. They were designed to complement individual motif finders for achieving better accuracy. The results can be clustered and ranked for obtaining the top significant motifs. Some pipelines allow verifying the results with the reference databases such as TRANSFAC [12], Jaspar [13], and UniPROBE [14] by using a motif comparison tool such as STAMP [15] or TOMTOM [16].

Table 1 gives a summary of some current pipelines. We briefly discuss some of their general limitations here. W-ChIPMotifs was designed for mouse and human species only. There is no option for running different combinations of motif finders in the pipeline. The results include the top ranked motifs and their matches from the reference database by using STAMP tool. GimmeMotifs is a standalone application that has several functions including motif finding. However, the results from motif discovery module only present the top ranked motifs and their matches in the reference database. CompleteMOTIFS allowed selecting the tools to run the motif discovery. The results showed the top ten predicted motifs from each selected tool and their matches in the reference database via STAMP tool. However, this pipeline is no longer available for use. MEME-ChIP reports the predicted motifs from each tool and their matches in the reference database by using TOMTOM. RSAT peak-motifs allows selecting the motif discovery algorithms and it reports the predicted motifs from each selected algorithm with their matches in the reference database. MotifLab is a standalone application with a wide-range of functions including motif discovery. As other pipelines, only the top ranked motifs are presented in the results. Promzea is specialized for maize, rice, and *Arabidopsis thaliana*. It presents only the top predicted motifs that are not verified with the reference database.

**Table 1:** Characteristics of some existing motif discovery pipelines

Pipeline	Components	Function	Input Format	Reference Database	Target Species	Platform	Year	Ref.
W-ChIPMotifs	Weeder, MaMF, Weeder, STAMP	Predict motifs from ChIP-Seq data	FASTA	TRANSFAC, Jaspar	Mouse Human	Web portal	2009	[5]
CompleteMOTIFS	MEME, Weeder, ChIPMunk, Patser, STAMP	Predict motifs from ChIP-Seq data	FASTA, BED, GFF	TRANSFAC, Jaspar, User-defined file	Unspecified	Web portal	2011	[7]
GimmeMotifs	BioProspector, GADEM, Improbizer, MDmodule, MEME, MoAn, MotifSampler, Trawler, Weeder	Predict motifs from ChIP-Seq data	BED, FASTA	Jaspar	Unspecified	Standalone application	2011	[6]
MEME-ChIP	MEME, DREME, CentriMo, TOMTOM, SpaMo	Predict motifs from ChIP-Seq data	FASTA	Jaspar, UniProbe, User-defined file, etc.…	Unspecified	Web portal, Web-services, Command line tool	2011	[8]
RSAT peak-motifs	Oligo-analysis, Position-analysis, Local-word analysis, Dyad-analysis	Predict motifs from ChIP-Seq data	FASTA	Jaspar, UniProbe, REGULONDB, User-defined file, etc.…	Unspecified	Web portal, Standalone application	2012	[9]
MotifLab	AlignAce, BioProspector, ChIPMunk, MEME, MotifSampler, Priority, Weeder	Analyze regulatory sequence regions, Predict binding site motifs	FASTA, BED, etc.…	TRANSFAC, Jaspar, ScerTF	Unspecified	Standalone application	2013	[10]
Promzea	BioProspector, MEME, Weeder, PSCAN, FIMO, Clover	Predict co-regulatory motifs	cDNA FASTA, microarray probe-set ID, BED	None	Maize, Rice, *Arabidopsis thaliana*	Web portal	2013	[11]

Although existing pipelines were designed with their unique integrations and the methods for ranking and selecting the significant motifs, they do not allow obtaining different comparison results for multiple tools and methods. They generally report the top ranked results either from individual motif finders or from a combination of multiple predictive algorithms and tools.

In this work, we incorporated four de novo motif finders namely ChIPMunk [17], MEME [18], Weeder [19], and XXmotif [20] into a pipeline called MODSIDE. The pipeline also integrated a motif similarity detection tool MOTIFSIM [21]. All adopted tools are open-source software. We chose ChIPMunk, MEME, and Weeder as they are widely used and some of their features are complemented. Since XXmotif is a general-purpose motif finder and it has some advanced features over three other motif finders, we adopted it for the pipeline. The features of these motif finders are presented in the Implementation section. We chose MOTIFSIM for similarity detection because of its unique features that are not offered by all existing pipelines. They include (1) the common (global) significant motifs from multiple tools, (2) the motifs detected by some tools but not by others (the global and local significant motifs), and (3) the best matches for each motif in the motif collection of multiple tools. Besides the unique features, MOTIFSIM also possesses other useful features for verifying the predicted motifs with the reference databases, merging similar motifs, and clustering predicted motifs into motif trees. MODSIDE pipeline delivers not only the results from individual motif finders but also the comparison results from the pipeline itself.

## Implementation

### Motif discovery

#### MEME

MEME (Multiple Expectation Maximization for Motif Elicitation) is a well-known motif discovery tool developed for targeting un-gapped motifs in unaligned DNA or protein sequences [18]. MEME algorithm is based on a profile-based method that implemented the expectation maximization (EM) [18]. The profile-based methods are faster than consensus-based methods but they suffer from lower accuracy because they tend to be trapped in a local optimum [22]. MEME algorithm removes the previous discovered motifs when it searches for new motifs. Thus, it can only model a single motif at a time and it does not detect alternative binding motifs, which are motifs for co-factors [23]. MEME also requires removing duplicate sequences and those with low information prior to running the tool [23]. Another drawback of MEME is splitting variable-length patterns into two or more separate motifs [18]. MEME was originally designed for discovering short motifs. However, its later versions allow finding longer motifs. MEME possesses numerous features for discovering motifs. These features are presented in the Additional file 1. We adopted version 4.11.4 for the pipeline.

#### ChIPMunk

ChIPMunk is a fast heuristic motif finder developed for analyzing high-throughput sequencing data [17]. ChIPMunk is also a profile-based method. Its algorithm implemented an iterative approach that combines the greedy optimization with bootstrapping. ChIPMunk evaluates the motif profiles based on the Kullback Discrete Information Content (KDIC). It employs a greedy approach for discovering the motif profiles with high KDIC values. The motif profiles are ranked based on Position Weight Matrix (PWM) scores. They are subsequently improved by an EM iterative process. ChIPMunk’s performance is better than MEME in term of runtime and prediction quality [24].

ChIPMunk was originally designed for discovering the motifs in PWMs for transcription factor binding sites. It was later adapted for handling ChIP-Seq data. ChIPMunk contains numerous attributes that are presented in the Additional file 1 for finding motifs. We adopted version 7 for the pipeline.

#### Weeder

Weeder was designed for finding DNA motifs [19]. Its algorithm is based on a pattern-driven approach, which is a sub-category of the consensus-based method [22]. Weeder algorithm implemented a suffix tree based exhaustive enumeration and extended it for searching longer patterns [19]. The algorithm was designed for finding subtle similarities in small datasets, rather than large similarities in large datasets [25]. Due to the nature of consensus-based method, Weeder is significantly slower than MEME and ChIPMunk but its prediction quality is higher. Weeder also comprises several attributes for finding DNA motifs. They are presented in the Additional file 1. We adopted version 1.4.2 for the pipeline.

#### XXmotif

XXmotif is a general-purpose method, which was designed for finding enriched motifs in nucleotide sequences [26]. However, unlike other motif finders, XXmotif is capable for optimizing the statistical significance of PWMs directly. It can also score conservation and positional clustering of motifs [20]. XXmotif algorithm is a combination of the pattern-based enumerative approach and the iterative PWM refinement [26]. The algorithm consists of masking stage, pattern stage, and PWM stage. The masking stage masks out the repeat regions, compositionally biased segments, and homologous segment pairs. The pattern stage calculates enrichment *P*-values of degenerate seed patterns. The PWM stage optimizes candidate PWMs iteratively [20]. The experimental results in [26] showed XXmotif has faster runtime and higher sensitivity than MEME and Weeder. In addition, the masking stage makes XXmotif more sophisticated, as this stage does not exist in MEME, ChIPMunk, and Weeder. As other tools, XXmotif provides a wide-range of features for finding motifs. We adopted its current version for the pipeline.

### Motif comparison

The pipeline incorporated MOTIFSIM version 2.2. MOTIFSIM allows comparing the results from multiple tools for attaining the common significant motifs, the motifs reported by some tools but not by others, as well as the best matches for each predicted motif in the motif collection for multiple tools. The results from multiple tools can be verified with the reference database such as TRANSFAC, Jaspar, or UniPROBE. Since the predicted motifs reported by a single tool or multiple tools can be redundant motifs. MOTIFSIM provides an option for merging them to reduce the number of redundant motifs. The new motif is formed only of it is within the similarity threshold with both of its parents [21]. Another useful feature of MOTIFSIM is clustering the motifs into motif trees. The tree describes the relationship between motifs. MOTIFSIM calculates the similarity scores between motifs and builds two distance matrices. One is for the global significant motifs and the other is for every motif in the motif collection of multiple tools. The distance matrices contain the best similarity scores between motifs. MOTIFSIM uses the distance matrices to build the motif trees by using *hclust* function in *R*. This function implemented the hierarchical clustering algorithm [21]. Hence, the motifs that reside in the same branch of the tree are more similar to one another. The height of the branch also shows the degree of similarity. The motifs that are connected by shorter branches are more similar than those that are connected by taller branches.

### MODSIDE web Interface

**Fig. 1:**
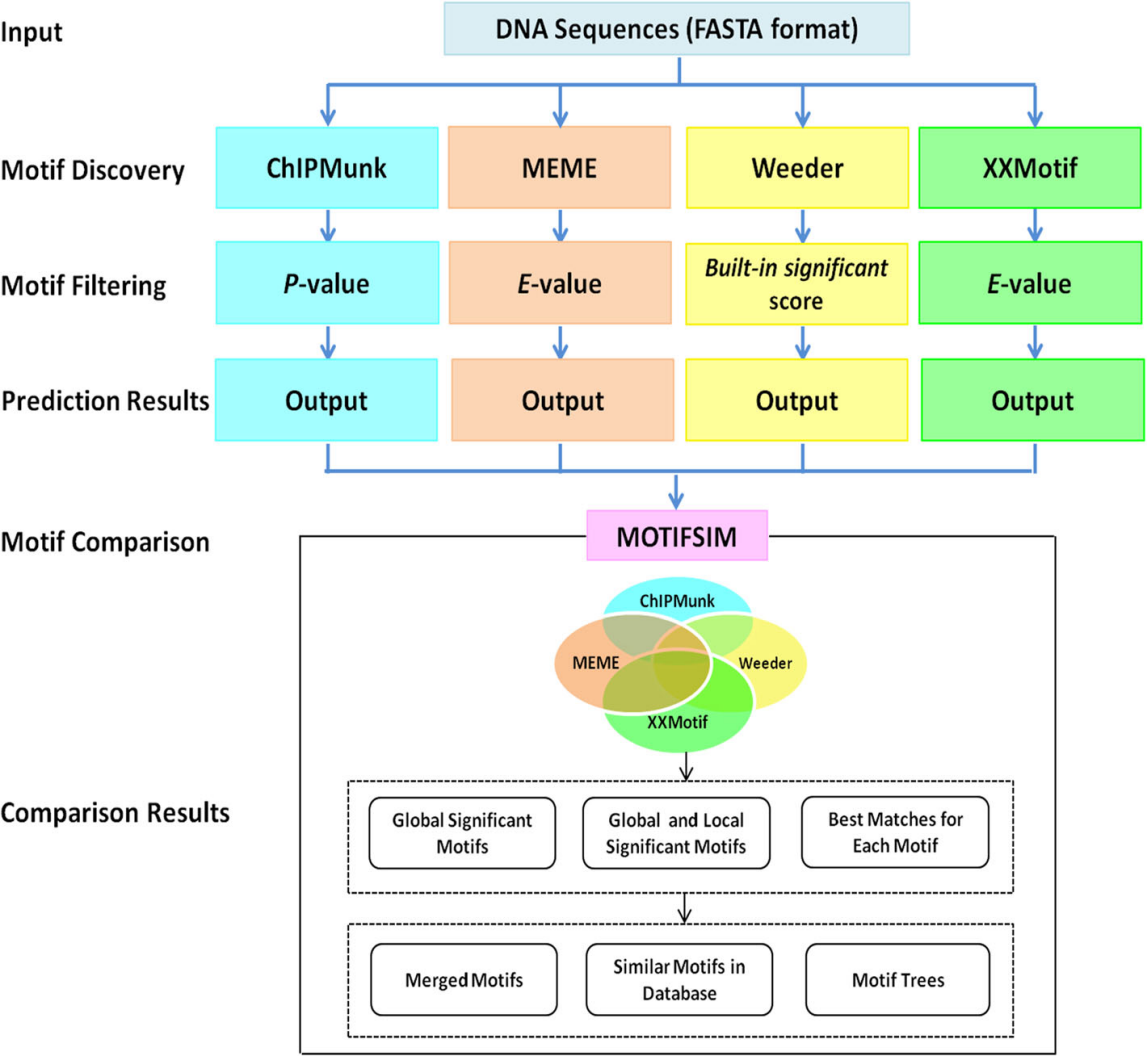
Workflow of MODSIDE. The pipeline takes DNA input sequences in FASTA format. The motif discovery module has ChIPMunk, MEME, Weeder, and XXmotif. They can be run in a combination of at least two tools. The significant motifs are selected by using *P*-value ≤0.05 for ChIPMunk, *E*-value ≤0.05 for MEME and XXmotif, and the built-in significant score in Weeder. The selected motifs are subsequently fed into MOTIFSIM for comparisons. The comparison results include the global (common) significant motifs, the global and local significant motifs, and the best matches for each motif in the motif collection of multiple tools. MOTIFSIM also provides the options for generating the motif trees, merging similar motifs, and verifying the predicted motifs with the reference database

MODSIDE was written in PHP, HTML, and JavaScript. The Web interface is publicly available at http://modside.org/. An overview of MODSIDE’s workflow is in Fig. 1. The pipeline accepts input in FASTA format. It can be run with at least two or more motif finders. The significant motifs are selected by using *P*-value ≤0.5 for ChIPMunk, *E*-value ≤0.5 for MEME and XXmotif, and the built-in significant score in Weeder. The descriptions for these thresholds are presented in the Additional file 1. The motif similarity detection and analysis module come from MOTIFSIM, which provides multiple options for comparing and analyzing the motifs. The options include the number of top significant motifs, the number of best matches, similarity cutoff, database matching, motif tree, and combining similar motifs. The results from individual motif finder are accessible for downloading and viewing. The comparison results from MOTIFSIM can be obtained in multiple formats. The job history can be retrieved by using Job ID via the Search Job page after the job is completed.

## Results

### Datasets

The pipeline was assessed on 16 benchmark sequence datasets from Tompa et al. in Table 2 [27]. They came from *Homo sapiens*, *Mus musculus*, and *Saccharomyces cerevisiae* species. The datasets can be *generic* or *Markov* type [27]. The generic type was generated by obtaining the promoter sequences randomly and implanted the known binding sites of the same species into those sequences. The Markov type was obtained by generating random sequences using Markov chain order of 3 and then implanted the known binding sites of the same species into those sequences. Each binding site embedded in a sequence belongs to a specific transcription factor in the TRANSFAC database. The transcription factor embedded in each sequence is listed in Table 2. We selected the benchmark datasets so that each sequence in a dataset has at least one or more embedded binding sites of the same transcription factor. These benchmarks were used to run MODSIDE with all motif finders selected. They were also used to run MEME-ChIP and RSAT peak-motifs.

**Table 2:** Sixteen benchmark sequence datasets [27]

Sequence Dataset	Dataset Type	Species	Transcription Factor	Number of Sequences	Sequence Length
hm01g	Generic	*Homo sapiens*	AP-1	18	2000
hm04g	Generic	*Homo sapiens*	c-Jun	13	2000
hm08m	Markov	*Homo sapiens*	CREB	15	500
hm15g	Generic	*Homo sapiens*	NF-1	4	2000
hm17g	Generic	*Homo sapiens*	NF-kappaB	11	500
hm19g	Generic	*Homo sapiens*	Sp1	5	500
hm22g	Generic	*Homo sapiens*	USF1	6	500
hm22m	Markov	*Homo sapiens*	USF1	6	500
mus09g	Generic	*Mus musculus*	POU2F1	2	500
mus10g	Generic	*Mus musculus*	Sp1	13	1000
mus11m	Markov	*Mus musculus*	Sp1	12	500
yst01g	Generic	*Saccharomyces cerevisiae*	ABF1	9	1000
yst02g	Generic	*Saccharomyces cerevisiae*	GAL04	4	500
yst03m	Markov	*Saccharomyces cerevisiae*	GCN4	8	500
yst06g	Generic	*Saccharomyces cerevisiae*	MCM1	7	500
yst09g	Generic	*Saccharomyces cerevisiae*	CAR1	16	1000

The datasets are grouped by species. Each dataset has a transcription factor embedded. Each dataset has different number of sequences and sequence length

### Evaluation

We evaluated MODSIDE in two phases. In the first phase, we assessed the accuracy of MODSIDE by comparing its results with the results from individual motif finder in the pipeline. The objective is to observe the efficiency of the pipeline and its motif finders. We used the assessment method, the benchmark sequence datasets, and the on-line assessment tool from Tompa et al. for this evaluation [27]. Tompa et al. introduced a comprehensive method for assessing computational tools for discovery of transcription factors binding sites. They built 52 benchmark datasets for evaluating 13 tools in their assessment. The technique used for creating these datasets was presented in the Datasets section. We employed six statistics from Tompa et al. for this evaluation. They are presented in the Additional file 1. The authors also built an assessment tool, which calculates several statistics including those used in this evaluation. The benchmark datasets and the assessment tool are available on-line. They can be used for assessing existing and future tools as well. We measured the accuracy of ChIPMunk, MEME, Weeder, XXmotif, and MODSIDE on 16 benchmark datasets. For each tool *T* and each dataset *D*, we have a set of known binding sites and a set of predicted binding sites. Thus, we can measure the accuracy of *T* on *D* at the nucleotide level and at the site level. At the nucleotide level, we calculated four statistics: Sensitivity (*nSn*), Positive Predictive Value (*nPPV*), Specificity (*nSP*), and Correlation coefficient (*nCC*). At the site level, we calculated two statistics that are Sensitivity (*sSn*) and Positive Predictive Value (*sPPV*). Since different tools produce different numbers of significant motifs by using the thresholds presented in the section MODSIDE Web Interface, we selected all significant motifs from each tool. We compared the significant motifs from these tools for the same sequence dataset by using MOTIFSIM for obtaining the global significant motifs [21]. Since MOTIFSIM identifies a set of common significant motifs reported by four tools, we selected the best common significant motif based on two criteria. First, it must represent the popular vote by majority of the tools. Second, it has the highest rank of similarity score. We assessed the accuracy of the top significant motif reported by each tool by using six statistics above. We then compared the accuracy for identifying the known motif of each tool including MODSIDE.

In the second phase, we compared MODSIDE with MEME-ChIP and RSAT peak-motifs for the following reasons. First, they are widely used. Second, they have no limitation for input species. Third, they have a user-friendly Web interface. Fourth, MEME-ChIP is based on a profile-based method, which has a lower accuracy while RSAT peak-motifs is based on a word-based method or consensus-based method, which has a higher accuracy. Hence, we expected to see RSAT peak-motifs outperforms MEME-ChIP. Alternatively, MODSIDE has a combination of both profile-based method and consensus-based method. This characteristic makes it interesting to observe the performance of each pipeline. In addition, all three pipelines have no limitation for input sequences as well as file size. Finally, like MODSIDE, both MEME-ChIP and RSAT peak-motifs have a feature for reporting the results of individual motif finders. Table 3 shows the characteristics of each pipeline.

**Table 3:** Characteristics of MEME-ChIP, RSAT peak-motifs, and MODSIDE

Pipeline	Components	Function	Input Format	Reference Database	Target Species	Sequence Limit	File Size Limit	Approach	Platform
MEME-ChIP	MEME, DREME, CentriMo, TOMTOM, SpaMo	Predict motifs from ChIP-Seq data	FASTA	Jaspar, UniProbe, User-defined file, etc.…	N/A	None	None	Profile-based method	Web portal, Web-services, Command line tool
RSAT peak-motifs	Oligo-analysis, Position-analysis, Local-word-analysis	Predict motifs from ChIP-Seq data	FASTA	Jaspar, UniProbe, REGULONDB, User-defined file, etc.…	N/A	None	None	Word-based method	Web portal, Standalone application
MODSIDE	ChIPMunk, MEME, Weeder, XXmotif, MOTIFSIM	Predict motifs in general and motifs from ChIP-Seq data Provide the common (global) significant motifs, the global and local significant motifs, the best matches for each motif in a combined motif list from multiple tools Merge similar motifs Generate motif tress	FASTA	Jaspar, TRANSFAC, UniPROBE	N/A	None	None	Profile-based method Consensus-based method	Web portal

We used the default setting provided by each pipeline to run the benchmark datasets in Table 2. The significant motifs were selected by using a similarity cut-off of ≥75% for MODSIDE and an *E*-value of ≤0.05 for MEME-ChIP and RSAT peak-motifs. We selected the top significant motif from each pipeline for each sequence dataset. We then calculated six statistics above for each top significant motif.

## Results

### MODSIDE versus ChIPMunk, MEME, Weeder, and XXmotif

**Fig. 2:**
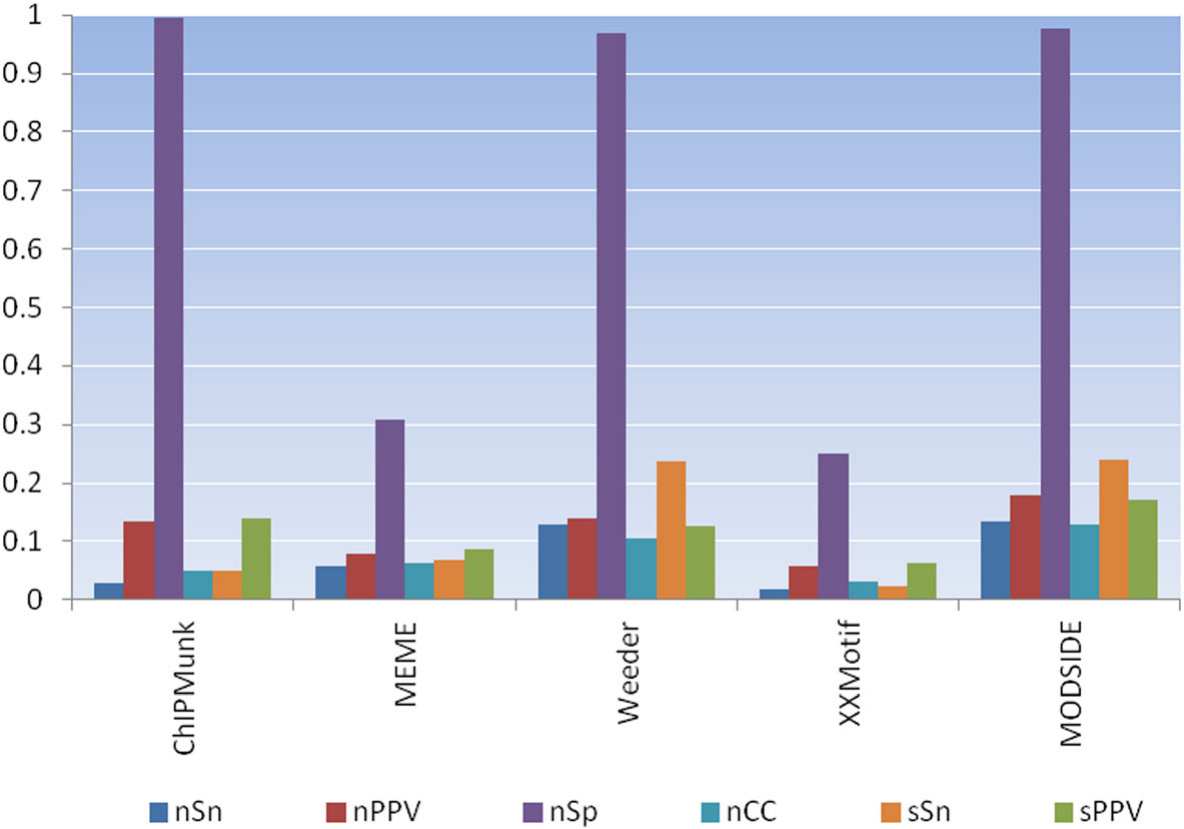
Average statistics for ChIPMunk, MEME, Weeder, XXmotif, and MODSIDE on sixteen benchmark datasets. Four statistics at the nucleotide level are Sensitivity (*nSn*), Positive Predictive Value (*nPPV*), Specificity (*nSp*), and Correlation Coefficient (*nCC*). Two statistics at the site level are Sensitivity (*sSn*) and Positive Predictive Value (*sPPV*) [27]. MODSIDE achieves better accuracy than other tools

We measured the accuracy of each tool by calculating six statistics in the Evaluation section for the top significant motif produced by each tool for the same sequence dataset. The results of four motif finders and MODSIDE on 16 benchmark datasets are in the Additional file 1: Figures S1-S16. The absent tools in the figures did not report any significant motif. They either failed to detect any motif or their reported motifs did not pass the significant threshold. This is due to the nature design and implementation of each tool. MEME and XXmotif did not report any significant motif for ten sequence datasets: *hm08m*, *hm19g*, *hm22g*, *hm22m*, *mus09g*, *mus11m*, *yst01g*, *yst02g*, *yst03m*, and *yst06g*. XXmotif failed to detect the known motif *NF-kappaB* although other tools identified it for sequence dataset *hm17g*. Besides, XXmotif and MEME did not report any significant motif for the sequence datasets *mus10g* and *yst09g* respectively. We calculated the average statistics for each tool including MODSIDE on 16 sequence datasets. The average result reveals MODSIDE attains better accuracy than individual motif finders. Figure 2 shows MODSIDE in the top rank followed by Weeder, MEME, ChIPMunk, and XXmotif respectively. The calculation can also be found in the Additional file 1: Table S1.

### MODSIDE versus MEME-ChIP and RSAT peak-motifs

**Fig. 3:**
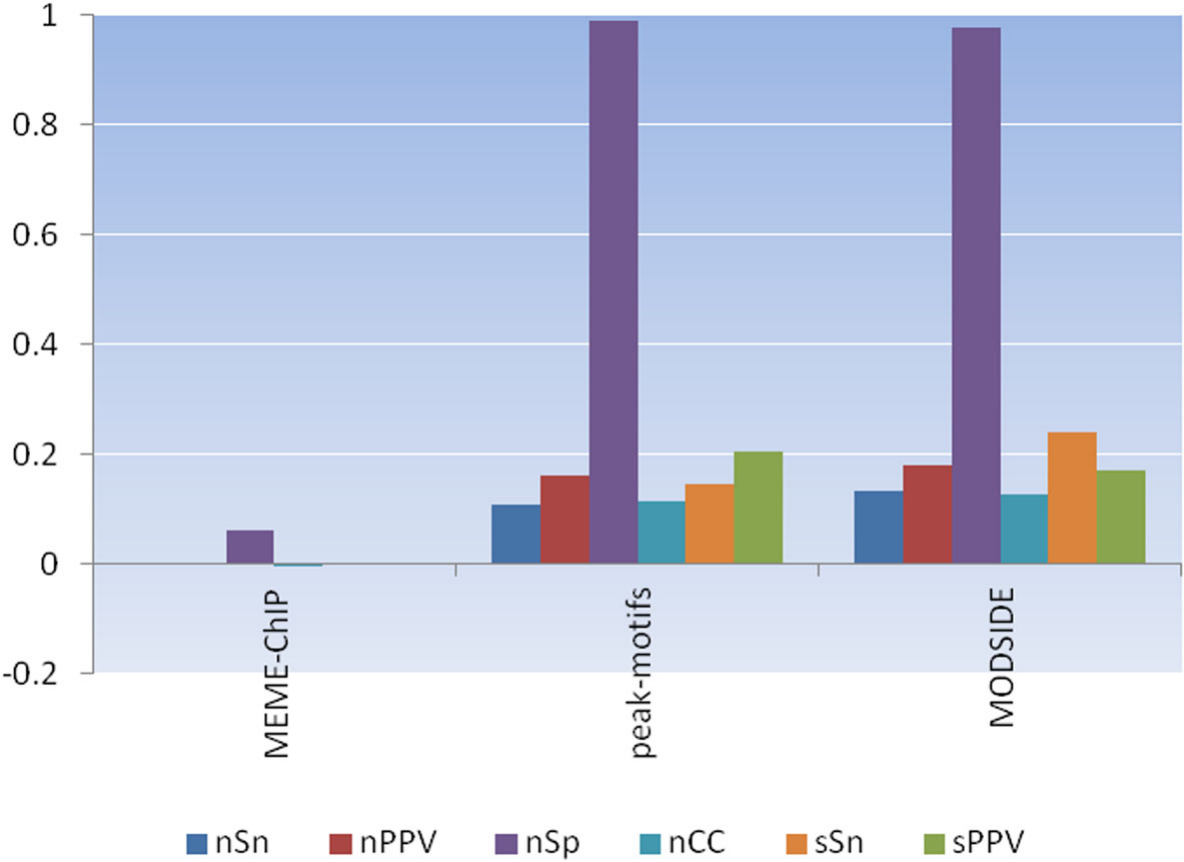
Average Statistics for MEME-ChIP, RSAT peak-motifs, and MODSIDE on sixteen benchmark datasets. MEME-ChIP has a lower accuracy than RSAT peak-motifs and MODSIDE. Both MODSIDE and RSAT peak-motifs achieve similar accuracy

We compared the accuracies of MEME-ChIP, RSAT peak-motifs, and MODSIDE by calculating six statistics for the top significant motif from each pipeline for each sequence dataset in Table 2. The statistical results are in Additional file 1: Figures S17-S32. Most of the figures do not show MEME-ChIP as it did not report any significant motif except for the dataset *hm04g* in Additional file 1: Figure S18. This is due to the nature design and implementation of MEME-ChIP and its components. All pipelines failed to identify the known motifs for the datasets *hm01g*, *hm04g*, *hm15g*, *hm22g*, *mus09g*, and *yst01g*. Again, this is due to the nature design and implementation of each pipeline and its components. For the rest of the datasets, either RSAT peak-motifs or MODSIDE can identify the known motifs with various degrees of accuracies. However, both RSAT peak-motifs and MODSIDE successfully identified the known motif *NF-kappaB* for the dataset *hm17g*. We calculated the average statistics for each pipeline on all sequence datasets as shown in Fig. 3 and in the Additional file 1: Table S2. MEME-ChIP shows a poorer accuracy than RSAT peak-motifs and MODSIDE. Again, this can be caused by the nature design and implementation of MEME-ChIP as presented above. However, both RSAT peak-motifs and MODSIDE expose a similar performance, as their average accuracies are quite similar. Nevertheless, MODSIDE has more advantages than MEME-ChIP and RSAT peak-motifs because it offers various comparison results that are not offered by MEME-ChIP, RSAT peak-motifs, and other existing pipelines.

## Conclusions

We developed MODSIDE for motif discovery and similarity detection. The pipeline delivers the predicted motifs from ChIPMunk, MEME, Weeder, and XXmotif. It also provides various comparison results for multiple motif finders. The comparison results include the common significant motifs, the motifs detected by some tools but not by others, as well as the best matches for each predicted motif in the collection of multiple tools. Besides, the pipeline allows comparing the predicted motifs with the reference databases for obtaining similar motifs. It also allows merging similar motifs and clustering the results into motif trees. We assessed MODSIDE and its motif finders on 16 benchmark datasets. The statistical results reveal MODSIDE attains better accuracy than its adopted motif finders. We also compared MODSIDE with MEME-ChIP and RSAT peak-motifs. The comparison results show MODSIDE and RSAT peak-motifs achieve similar performance while MEME-ChIP has a lower accuracy than other two pipelines. Although the performance of MODSIDE is comparable to RSAT peak-motifs, it offers various comparison results that are not offered by RSAT peak-motifs and other existing motif discovery pipelines.

## Availability and requirements

Project name: modside

Project home page: http://modside.org/

Operating system(s): Linux

Programming language: C++, PHP, JavaScript, Python, and R

Other requirements: Apache2 Web server, open source Prince software package, and WebLogo v. 3.4

License: GNU

Any restrictions to use by non-academics: None

## Additional file

Additional file 1: Supplementary Materials. (DOCX 726 kb)

## Abbreviations

EM: Expectation maximization
KDIC: Kullback Discrete Information Content
PWM: Position Weight Matrix
