# Supplementary material for: MODSIDE: a motif discovery pipeline and similarity detector
Source: BMC Genomics. 2018 Oct 19;19:755. doi: 10.1186/s12864-018-5148-1 (PMC6194616; doi:10.1186/s12864-018-5148-1)
Supplement: Supplementary file 1 — Supplementary Materials. (DOCX 726 kb) [file 12864_2018_5148_MOESM1_ESM.docx]

### MODSIDE: A Motif Discovery Pipeline and Similarity Detector

Ngoc Tam L. Tran and Chun-Hsi Huang

### SUPPLEMENTARY MATERIALS

**Motif Finders and Features**

***MEME***

MEME possesses numerous features for discovering motifs. The motif discovery mode currently provides three options including classic, discriminative, and differential enrichment modes. MEME accepts different input sequence types such as DNA, RNA, and protein. The site distribution feature provides three options for specifying the occurrences of the motifs. They include zero or one occurrence per sequence, one occurrence per sequence, and any number of repetitions. Other features that can be specified include the number of discovered motifs, the background model, motif length, the number of motif sites for each motif, searching direction for DNA and RNA strands, limiting the search to palindromes, and shuffling the sequences [1].

***ChIPMunk***

ChIPMunk has numerous attributes for finding motifs. The Mode attribute allows specifying different types of input sequences such as basic sequences, sequences with prior reliability data, sequences with positional prior data, and single-stranded mode for RNA motif discovery. ChIPMunk can take input sequences in extended multifasta format. The Speed mode attribute provides a fast mode for quickly checking the data for putative pattern and a precise mode, which is a detailed analysis for providing more robust results. Like MEME, ChIPMunk also allows specifying the occurrences of motif per sequence such as one occurrence per sequence and zero or one occurrence per sequence. Other attributes include motif length, motif shape, and background model [2].

***Weeder***

Weeder comprises several attributes for finding DNA motifs. These attributes include organism, motif length, searching direction for DNA strand, occurrences of motifs in the sequences, and highest scoring motifs of each run [3].

***XXmotif***

XXmotif also has a wide-range of features for finding motifs. These features include input format, background model, occurrences of motifs per sequence, searching direction for DNA strand, order of background-model, similarity threshold for merging motifs/PWMs, types of seed patterns, number of gaps in seed, pseudocounts, number of sequences of alignments, and masking option for input set [4].

**Statistics used in Evaluation**

Tompa *et al.* measured the correctness of a tool *T* on a dataset *D* at both *nucleotide level* and at *site level*. At the nucleotide level, true positives (*nTP*), false negatives (*nFN*), false positive (*nFP*), and true negative (*nTN*) are defined as follows [5].

- *nTP*: the number of nucleotide positions in both known sites and predicted sites.
- *nFN*: the number of nucleotide positions in known sites but not in predicted sites.
- *nFP*: the number of nucleotide positions not in known sites but in predicted sites.
- *nTN*: the number of nucleotide positions in neither known sites nor predicted sites.

At the site level, a predicted site is considered overlapping a known site if they overlap by at least one-quarter the length of the known site. Similarly, true positives (*sTP*), false negatives (*sFN*), and false positives (*sFP*) are also defined as follows [5].

- *sTP*: the number of known sites overlapped by predicted sites.
- *sFN*: the number of known sites not overlapped by predicted sites.
- *sFP*: the number of predicted sites not overlapped by known sites.

Further, at nucleotide level (*x* = *n*) or site level (*x* = *s*), six statistics used in this validation are defined as follows [5].

- *Sensitivity*: $xSn=\frac{xTP}{\left( xTP+xFN \right)}$

*xSn* gives the portion of known sites that are predicted.

- *Positive Predictive Value*: $xPPV=\frac{xTP}{\left( xTP+xFP \right)}$

*xPPV* gives the portion of predicted sites that are known.

- *Specificity*: $nSP=\frac{nTN}{\left( nTN+nFP \right)}$

*nSP* gives the portion of nucleotides that are known neither in known sites nor in predicted sites.

- *Correlation Coefficient*:

$$nCC=\frac{\left( nTP\times nTN \right)-\left( nFN\times nFP \right)}{\sqrt{\left( nTP+nFN \right)\left( nTN+nFP \right)\left( nTP+nFP \right)\left( nTN+nFN \right)}}$$

*nCC* is the Pearson product-moment coefficient of correlation. It measures the correlation between two sets of positions, which are the known nucleotide positions and the predicted nucleotide positions. *nCC* has the value ranging from -1 for perfect no correlation to +1 for perfect correlation. If the predicted motifs exactly match with the known binding sites, then *nCC* would be +1. Otherwise, if each nucleotide position was predicted to be in the motif randomly and independently, then the expected value of *nCC* would be 0 for no correlation [5].

***P*-value**

The *P*-value is the probability to reject the null hypothesis called $H_{0}$ of a study question when the hypothesis is true. The null hypothesis is usually a hypothesis assumes that there is no difference between two populations. In motif discovery case, the null hypothesis usually assumes that there is no difference between the predicted motif and the background model. The alternative hypothesis called $H_{1}$ is the opposite of the null hypothesis and it is usually the one under examination. In motif discovery case, the alternative hypothesis assumes that there is a difference between the predicted motif and the background model. In other words, the predicted motif is over-represented in the data and it is not the same as the background model. Usually, a threshold is set for selecting the *P*-value. The common thresholds are 0.05 and 0.01. If the *P*-value is less or equals to the threshold, the null hypothesis is rejected. In this case, it means that the predicted motif is over-represented and it is not the same as the background model.

***E*-value**

The *E*-value is the expected value or the correction of *P*-value in multiple testing. For small *E*-value ≤ 0.01, *P*-value and *E*-value are almost identical.

Most of motif finders calculate the *P*-value or *E*-value for the predicted motif. We chose a threshold of 0.05 or less for both *P*-value and *E*-value for selecting the significant motifs from ChIPMunk, MEME, and XXmotif.

**Significant Score in Weeder**

Weeder uses its own method for calculating the significant scores for the significant motifs that the tool predicted [3]. Since this score was calculated at runtime by the tool, we only selected all significant motifs generated by the tool. The authors call them “interesting motifs” in the result file.

**Figures**

| 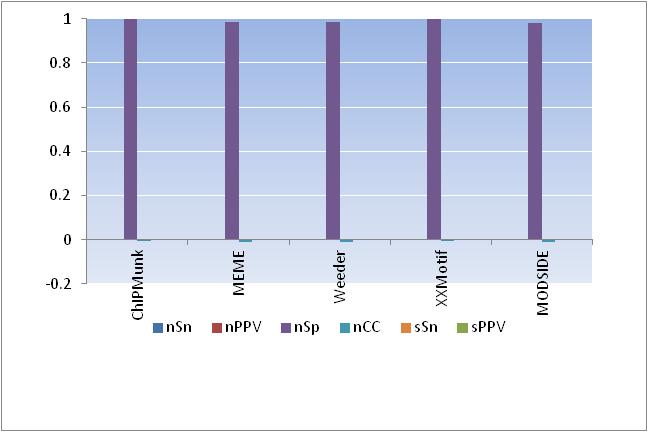  **Figure S1. Performance comparison for four motif finders and MODSIDE on dataset hm01g. The selected global significant motif (best match for this dataset) from MODSIDE is Motif4 came from Weeder.** | 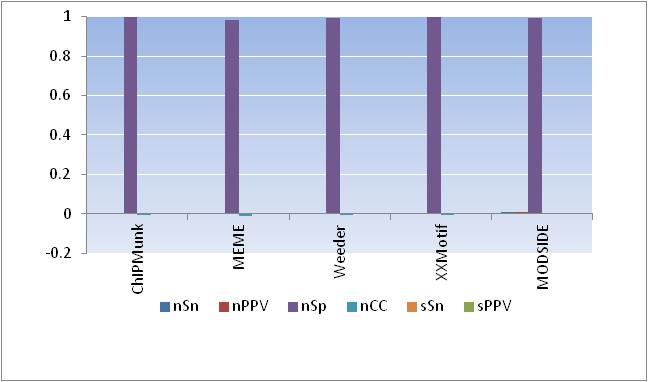  **Figure S2. Performance comparison for four motif finders and MODSIDE on dataset hm04g. The selected global significant motif (best match for this dataset) from MODSIDE is Motif3 came from Weeder.** |
| --- | --- |
| 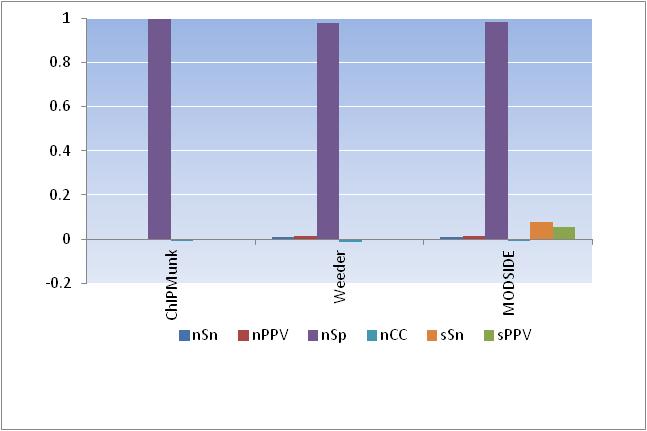  **Figure S3. Performance comparison for ChIPMunk, Weeder, and MODSIDE on dataset hm08m. MEME and XXmotif did not report any significant motif. The selected global significant motif (best match for this dataset) from MODSIDE is Motif7 came from Weeder.** | 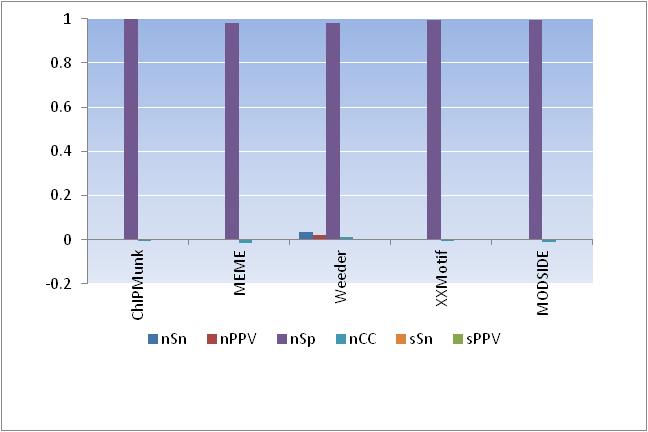  **Figure S4. Performance comparison for four motif finders and MODSIDE on dataset hm15g. The selected global significant motif (best match for this dataset) from MODSIDE is Motif7 came from Weeder.** |
| 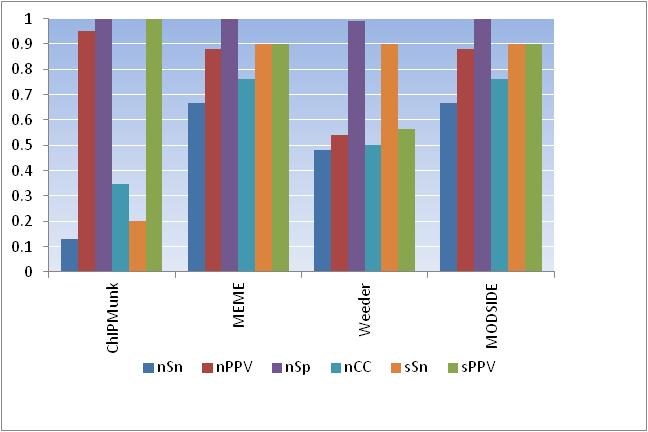  **Figure S5. Performance comparison for ChIPMunk, MEME, Weeder, and MODSIDE on dataset hm17g. XXmotif did not report any significant motif. The selected global significant motif (best match for this dataset) from MODSIDE is Motif5 came from MEME.** | 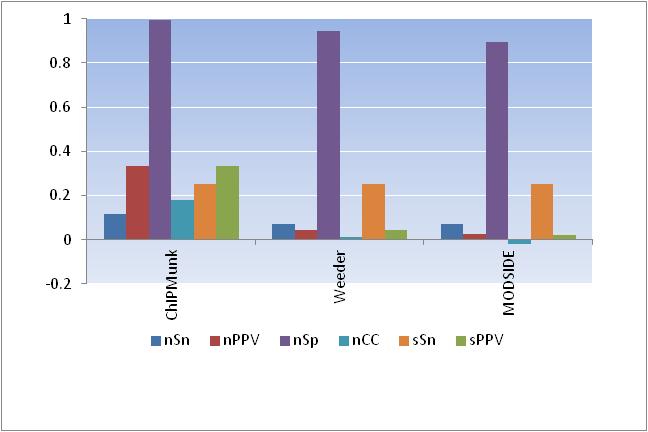  **Figure S6. Performance comparison for ChIPMunk, Weeder, and MODSIDE on dataset hm19g. MEME and XXmotif did not report any significant motif. The selected global significant motif (best match for this dataset) from MODSIDE is Motif7 came from Weeder.** |
| 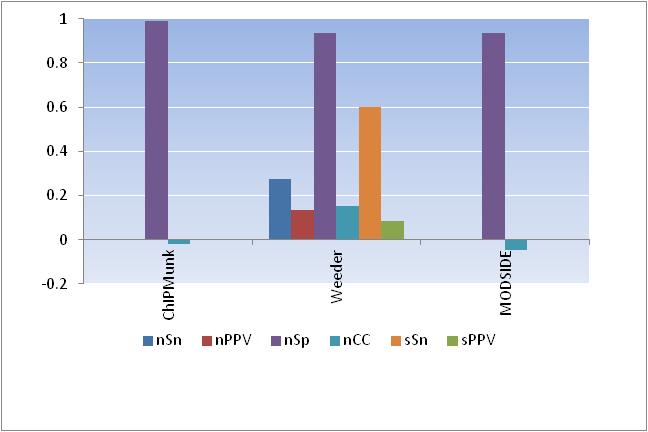  **Figure S7. Performance comparison for ChIPMunk, Weeder, and MODSIDE on dataset hm22g. MEME and XXmotif did not report any significant motif. The selected global significant motif (best match for this dataset) from MODSIDE is Motif7 came from Weeder.** | 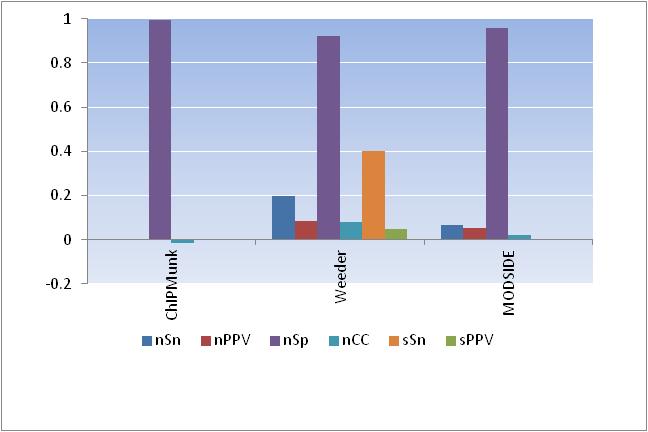  **Figure S8. Performance comparison for ChIPMunk, Weeder, and MODSIDE on dataset hm22m. MEME and XXmotif did not report any significant motif. The selected global significant motif (best match for this dataset) from MODSIDE is Motif5 came from Weeder.** |
| 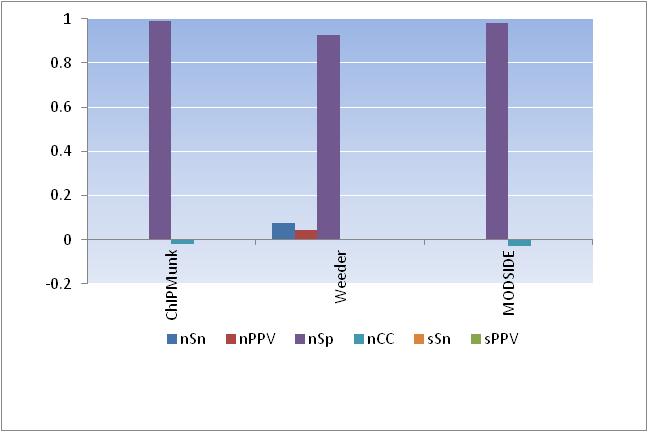  **Figure S9. Performance comparison for ChIPMunk, Weeder, and MODSIDE on dataset mus09g. MEME and XXmotif did not report any significant motif. The selected global significant motif (best match for this dataset) from MODSIDE is Motif6 came from Weeder.** | 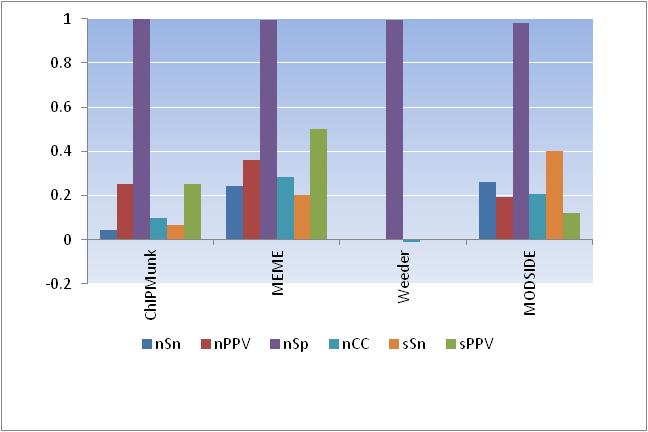  **Figure S10. Performance comparison for ChIPMunk, MEME, Weeder, and MODSIDE on dataset mus10g. XXmotif did not report any significant motif. The selected global significant motif (best match for this dataset) from MODSIDE is Motif4 came from Weeder.** |
| 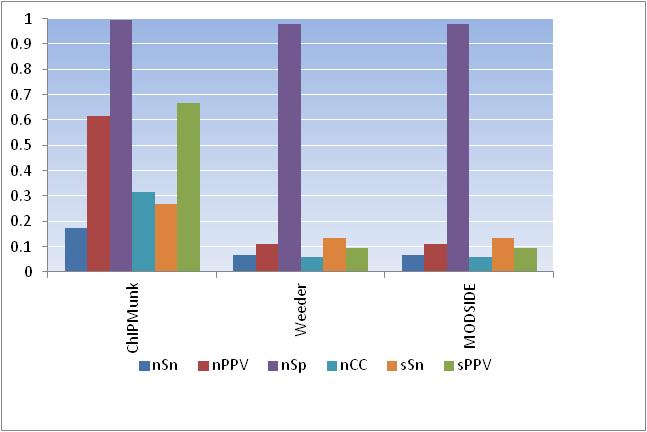  **Figure S11. Performance comparison for ChIPMunk, Weeder, and MODSIDE on dataset mus11m. MEME and XXmotif did not report any significant motif. The selected global significant motif (best match for this dataset) from MODSIDE is Motif1 came from Weeder.** | 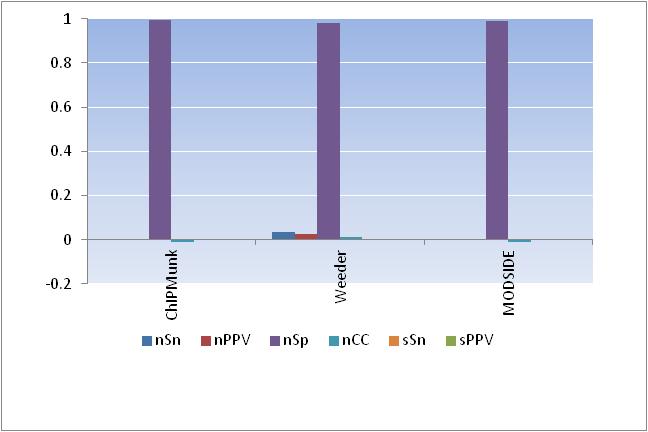  **Figure S12. Performance comparison for ChIPMunk, Weeder, and MODSIDE on dataset yst01g. MEME and XXmotif did not report any significant motif. The selected global significant motif (best match for this dataset) from MODSIDE is Motif3 came from Weeder.** |
| 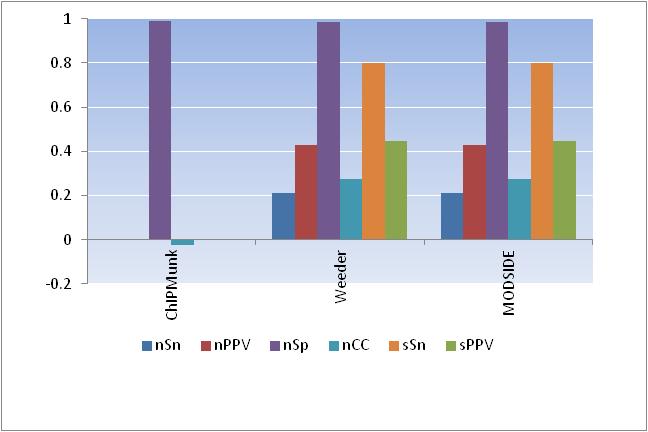  **Figure S13. Performance comparison for ChIPMunk, Weeder, and MODSIDE on dataset yst02g. MEME and XXmotif did not report any significant motif. The selected global significant motif (best match for this dataset) from MODSIDE is Motif1 came from Weeder.** | 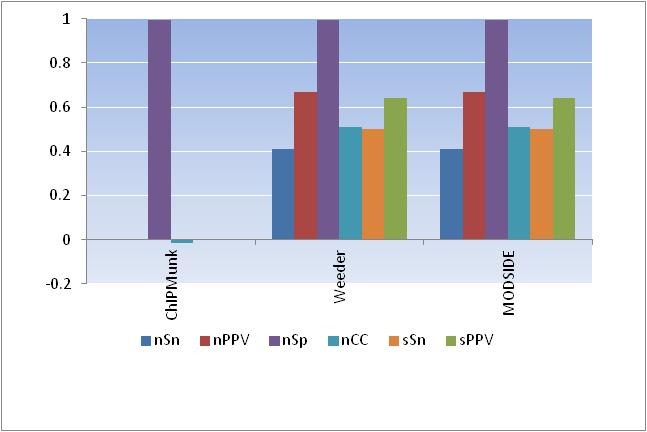  **Figure S14. Performance comparison for ChIPMunk, Weeder, and MODSIDE on dataset yst03m. MEME and XXmotif did not report any significant motif. The selected global significant motif (best match for this dataset) from MODSIDE is Motif1 came from Weeder.** |
| 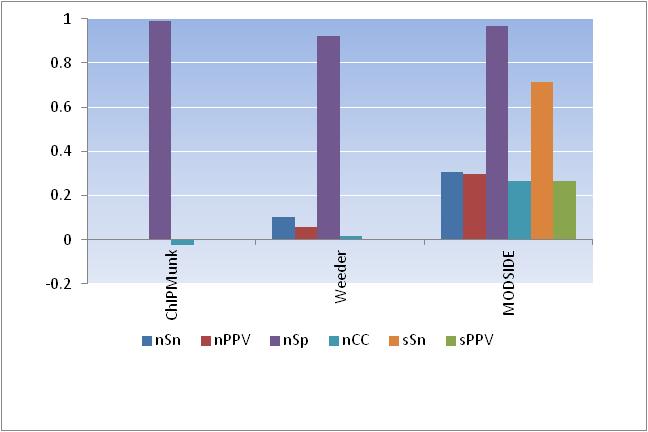  **Figure S15. Performance comparison for ChIPMunk, Weeder, and MODSIDE on dataset yst06g. MEME and XXmotif did not report any significant motif. The selected global significant motif (best match for this dataset) from MODSIDE is Motif6 came from Weeder.** | 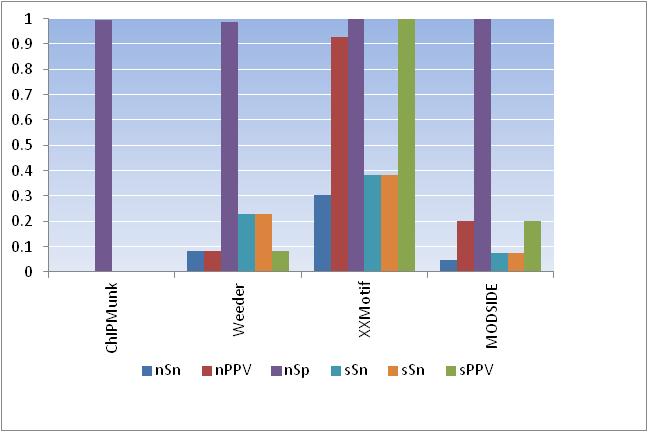  **Figure S16. Performance comparison for ChIPMunk, Weeder, XXmotif, and MODSIDE on dataset yst09g. MEME did not report any significant motif. The selected global significant motif (best match for this dataset) from MODSIDE is Motif6 came from Weeder.** |
| 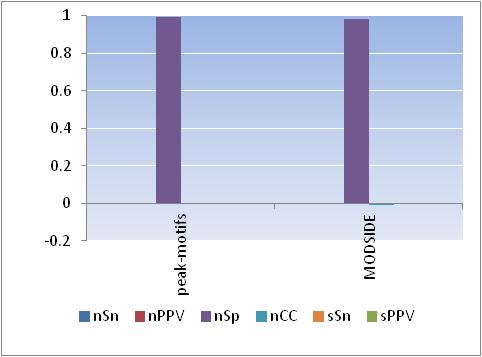  **Figure S17. Performance comparison for RSAT peak-motifs and MODSIDE on dataset hm01g. MEME-ChIP did not report any significant motif.** | 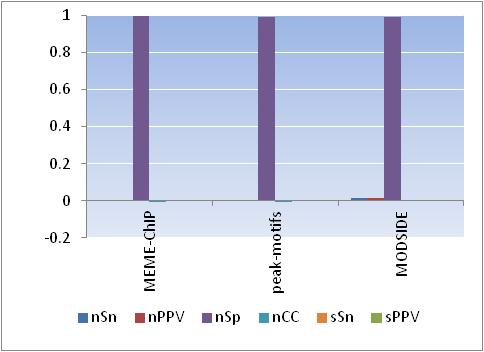  **Figure S18. Performance comparison for MEME-ChIP, RSAT peak-motifs, and MODSIDE on dataset hm04g.** |
| 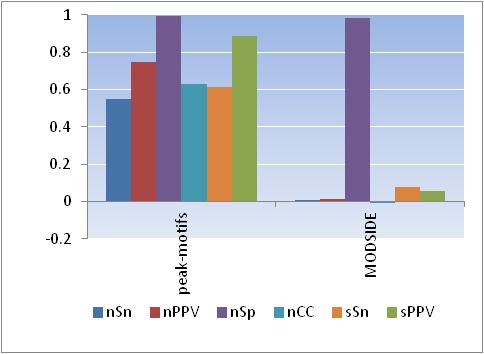  **Figure S19. Performance comparison for RSAT peak-motifs and MODSIDE on dataset hm08m. MEME-ChIP did not report any significant motif.** | 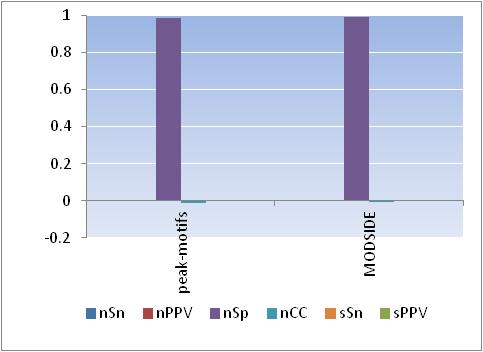  **Figure S20. Performance comparison for RSAT peak-motifs and MODSIDE on dataset hm15g. MEME-ChIP did not report any significant motif.** |
| 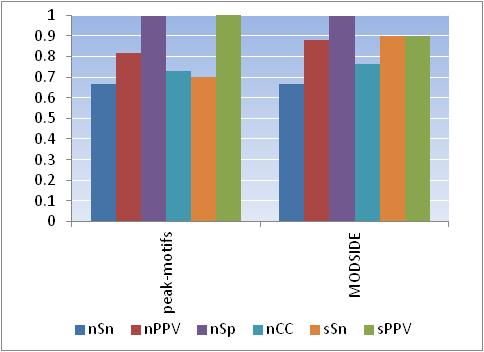  **Figure S21. Performance comparison for RSAT peak-motifs and MODSIDE on dataset hm17g. MEME-ChIP did not report any significant motif.** | 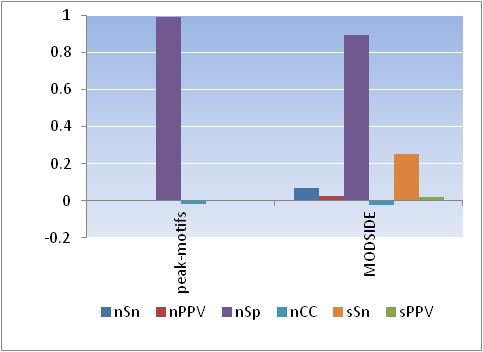  **Figure S22. Performance comparison for RSAT peak-motifs and MODSIDE on dataset hm19g. MEME-ChIP did not report any significant motif.** |
| 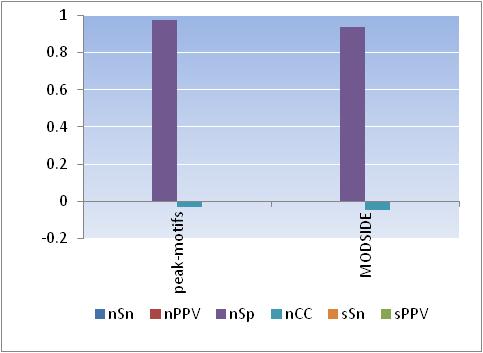  **Figure S23. Performance comparison for RSAT peak-motifs and MODSIDE on dataset hm22g. MEME-ChIP did not report any significant motif.** | 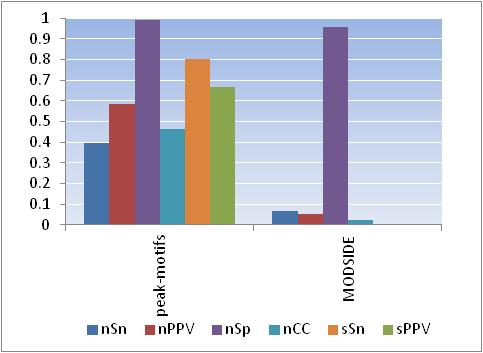  **Figure S24. Performance comparison for RSAT peak-motifs and MODSIDE on dataset hm22m. MEME-ChIP did not report any significant motif.** |
| 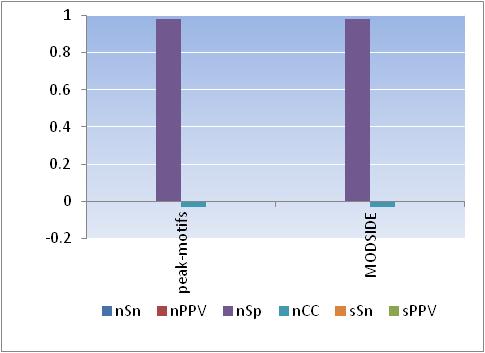  **Figure S25. Performance comparison for RSAT peak-motifs and MODSIDE on dataset mus09g. MEME-ChIP did not report any significant motif.** | 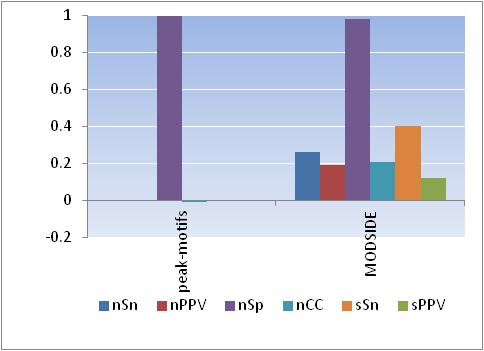  **Figure S26. Performance comparison for RSAT peak-motifs and MODSIDE on dataset mus10g. MEME-ChIP did not report any significant motif.** |
| 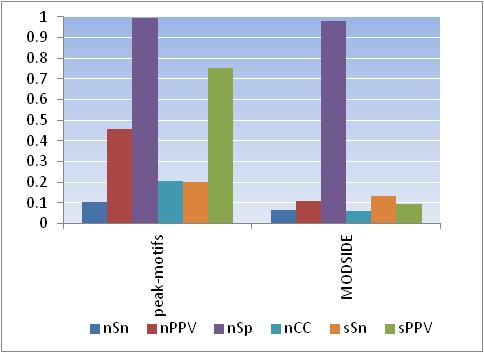  **Figure S27. Performance comparison for RSAT peak-motifs and MODSIDE on dataset mus11m. MEME-ChIP did not report any significant motif.** | 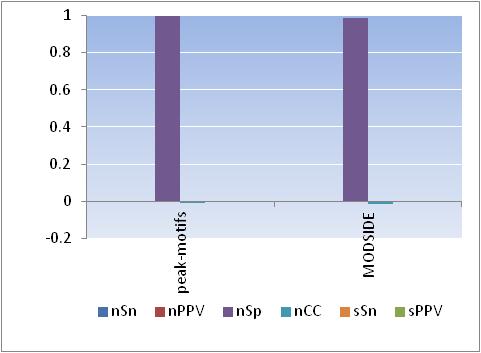  **Figure S28. Performance comparison for RSAT peak-motifs and MODSIDE on dataset yst01g. MEME-ChIP did not report any significant motif.** |
| 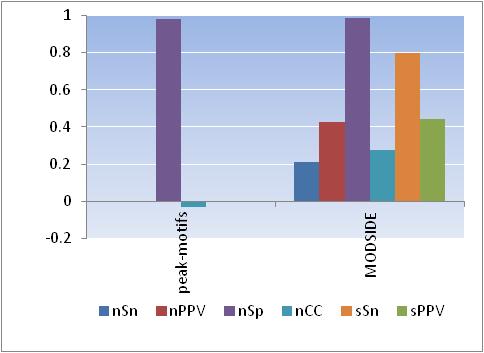  **Figure S29. Performance comparison for RSAT peak-motifs and MODSIDE on dataset yst02g. MEME-ChIP did not report any significant motif.** | 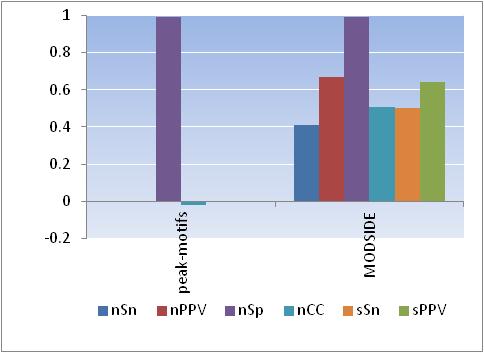  **Figure S30. Performance comparison for RSAT peak-motifs and MODSIDE on dataset yst03m. MEME-ChIP did not report any significant motif.** |
| 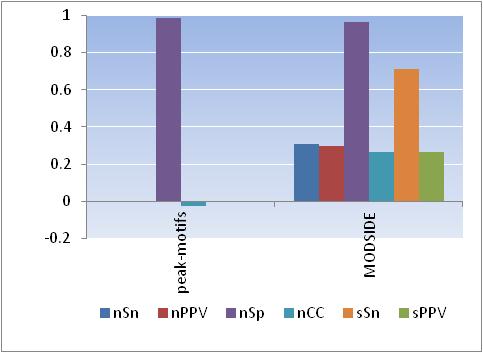  **Figure S31. Performance comparison for RSAT peak-motifs and MODSIDE on dataset yst06g. MEME-ChIP did not report any significant motif.** | 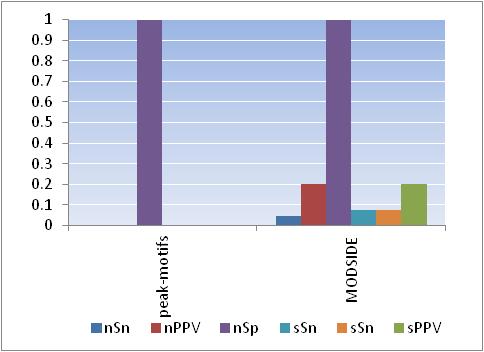  **Figure S32. Performance comparison for RSAT peak-motifs and MODSIDE on dataset yst09g. MEME-ChIP did not report any significant motif.** |

**Tables**

**Table S1. Average statistics for ChIPMunk, MEME, Weeder, XXmotif, and MODSIDE on sixteen benchmark datasets.** Four statistics at the nucleotide level are Sensitivity (*nSn*), Positive Predictive Value (*nPPV*), Specificity (*nSp*), and Correlation Coefficient (*nCC*). Two statistics at the site level are Sensitivity (*sSn*) and Positive Predictive Value (*sPPV*) [5].

| **Tool Name** | ***nSn*** | ***nPPV*** | ***nSp*** | ***nCC*** | ***sSn*** | ***sPPV*** |
| --- | --- | --- | --- | --- | --- | --- |
| ChIPMunk | 0.0291 | 0.1344 | 0.9943 | 0.0493 | 0.0490 | 0.1406 |
| MEME | 0.0569 | 0.0776 | 0.3087 | 0.0633 | 0.0688 | 0.0875 |
| Weeder | 0.1278 | 0.1406 | 0.9690 | 0.1038 | 0.2384 | 0.1254 |
| XXMotif | 0.0189 | 0.0580 | 0.2493 | 0.0320 | 0.0240 | 0.0625 |
| MODSIDE | 0.1329 | 0.1798 | 0.9754 | 0.1285 | 0.2407 | 0.1714 |

**Table S2. Average statistics for MEME-ChIP, RSAT peak-motifs, and MODSIDE on sixteen benchmark datasets.**

| **Tool Name** | ***nSn*** | ***nPPV*** | ***nSp*** | ***nCC*** | ***sSn*** | ***sPPV*** |
| --- | --- | --- | --- | --- | --- | --- |
| MEME-ChIP | 0.0000 | 0.0000 | 0.0622 | -0.0003 | 0.0000 | 0.0000 |
| RSAT peak-motifs | 0.1073 | 0.1628 | 0.9903 | 0.1150 | 0.1447 | 0.2066 |
| MODSIDE | 0.1329 | 0.1798 | 0.9754 | 0.1285 | 0.2407 | 0.1714 |

**References**

1. Bailey T, Williams N, Misleh C, Li W. MEME: discovering and analyzing DNA and protein sequence motifs. Nucleic Acids Res. 2006;34(Web Server issue):W369-W373.
2. Kulakovskiy IV, Boeva VA, Favorov AV, Makeev VJ. Deep and wide digging for binding motifs in ChIP-Seq data. Bioinformatics. 2010;26(20):2622-3.
3. Pavesi G, Mauri G, Pesole G. An algorithm for finding signals of unknown length in DNA sequences. Bioinformatics. 2001;17:S207-14.
4. Luehr S, Hartmann H, Söding J. The XXmotif web server for eXhaustive, weight matriX-based motif discovery in nucleotide sequences. Nucleic Acids Res. 2012;40(Web Server issue):W104-9.
5. Tompa M, Li N, Bailey TL, Church GM, De Moor B, Eskin E et al. Assessing computational tools for the discovery of transcription factor binding sites. Nat Biotechnol. 2005;23(1):137-44.
